# TonB-Dependent Receptor Repertoire of Pseudomonas aeruginosa for Uptake of Siderophore-Drug Conjugates

**DOI:** 10.1128/AAC.00097-18

**Published:** 2018-05-25

**Authors:** Alexandre Luscher, Lucile Moynié, Pamela Saint Auguste, Dirk Bumann, Lena Mazza, Daniel Pletzer, James H. Naismith, Thilo Köhler

**Affiliations:** a Service of Infectious Diseases, University Hospital Geneva, Geneva, Switzerland; b Department of Microbiology and Molecular Medicine, University of Geneva, Geneva, Switzerland; c School of Chemistry and Biomedical Sciences Research Complex, University of St Andrews, Fife, Scotland, United Kingdom; d Biozentrum, University of Basel, Basel, Switzerland; e Jacobs University, Bremen, Germany

**Keywords:** Pseudomonas aeruginosa, TonB-dependent receptor, siderophore-drug conjugate

## Abstract

The conjugation of siderophores to antimicrobial molecules is an attractive strategy to overcome the low outer membrane permeability of Gram-negative bacteria. In this Trojan horse approach, the transport of drug conjugates is redirected via TonB-dependent receptors (TBDR), which are involved in the uptake of essential nutrients, including iron. Previous reports have demonstrated the involvement of the TBDRs PiuA and PirA from Pseudomonas aeruginosa and their orthologues in Acinetobacter baumannii in the uptake of siderophore-beta-lactam drug conjugates. By *in silico* screening, we further identified a PiuA orthologue, termed PiuD, present in clinical isolates, including strain LESB58. The *piuD* gene in LESB58 is located at the same genetic locus as *piuA* in strain PAO1. PiuD has a similar crystal structure as PiuA and is involved in the transport of the siderophore-drug conjugates BAL30072, MC-1, and cefiderocol in strain LESB58. To screen for additional siderophore-drug uptake systems, we overexpressed 28 of the 34 TBDRs of strain PAO1 and identified PfuA, OptE, OptJ, and the pyochelin receptor FptA as novel TBDRs conferring increased susceptibility to siderophore-drug conjugates. The existence of a TBDR repertoire in P. aeruginosa able to transport siderophore-drug molecules potentially decreases the likelihood of resistance emergence during therapy.

## INTRODUCTION

With the shortage of novel classes of antimicrobials, alternative approaches aiming to increase antimicrobial penetration into Gram-negative bacteria have gained widespread interest. Such approaches include the inhibition of broad-spectrum efflux pumps (1), adjuvants that increase cell permeability (2), and the redirection of drug uptake through specific nutrient transport systems (3). The most prominent example of the latter approach is the hijacking of essential bacterial iron transport systems by linking antimicrobial molecules to siderophores in a Trojan horse strategy. The recent development of such compounds by all major pharmaceutical companies historically involved in antimicrobial drug development highlights the increasing interest in this appealing concept (4–7). So far, most of the efforts have focused on the design and study of beta-lactam-siderophore conjugates. Since their targets are located in the periplasmic space, the conjugates do not require further translocation across the inner membrane. Moreover, the conjugates are designed such that the siderophore moiety does not interfere with the drug target interaction and does not require prior cleavage (8). The beta-lactam scaffolds used for the design of such conjugates include penicillins (9), cephalosporins (KP736 and cefiderocol) (7, 10), and monobactams (BAL30072 and MC-1) (4, 5). The iron-binding moiety of these beta-lactam conjugates is either a catechol-type siderophore such as dihydroxypyridone or a mixed catechol/hydroxamate (11). Both the monobactam (5, 12–14) and the cephalosporin conjugates (15) showed potent activity against the Gram-negative nonfermenters Pseudomonas aeruginosa and Acinetobacter baumannii.

Two TonB-dependent receptors (TBDRs), PiuA and PirA, have been shown to be responsible for the uptake of BAL30072, MC-1, and cefiderocol in P. aeruginosa (5, 16, 17). We previously observed that some P. aeruginosa isolates did not carry the *piuA* gene, although they were susceptible to BAL30072 (16). Therefore, we suspected that other TonB-dependent receptors (TBDRs) might be present in these strains or that their expression differs with respect to the PAO1 reference strain. Furthermore, the expression of TBDRs is often regulated by sigma/anti-sigma factors or two-component systems (18) and is induced by the presence of the corresponding siderophore (19, 20). These receptors could potentially participate in siderophore-drug uptake, but their contribution is masked under standard noninducing conditions. Therefore, we performed an *in silico* screen for PiuA orthologues in the P. aeruginosa genome database, and we additionally expressed from plasmids 28 of the 34 TBDRs of PAO1. This enabled us to identify a novel TBDR, termed PiuD, sharing 60% amino acid identity with PiuA, as well as five additional TBDRs of PAO1, potentially involved in the uptake of three different siderophore-drug conjugates, including the most recent catechol-based compound, cefiderocol (21).

## RESULTS

### *piuD* and *piuA* encode homologous proteins and are mutually exclusive in P. aeruginosa genomes.

**FIG 1:**
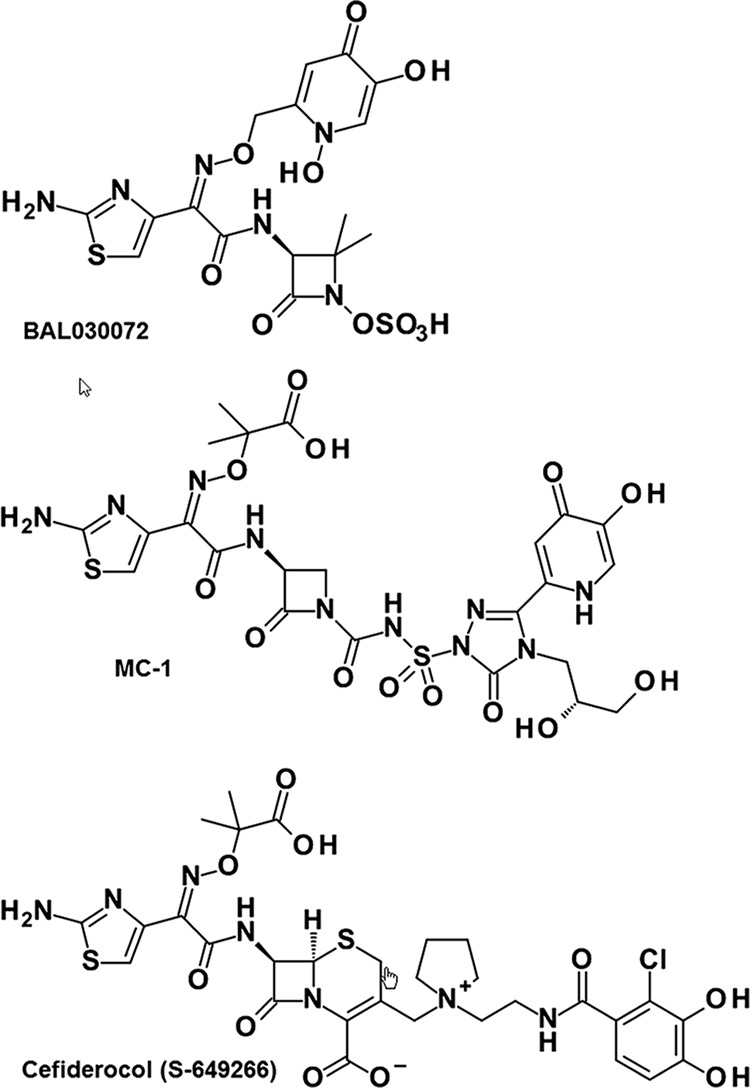
Siderophore-drug conjugates used in this study. BAL30072 and MC-1 contain a dihydroxypyridone as a siderophore, while cefiderocol contains a chlorinated catechol group.

We and others (5) previously identified the TonB-dependent receptors (TBDR) PiuA and PirA as transporters for the uptake of siderophore-drug conjugates BAL30072 and MC-1 (Fig. 1) both in P. aeruginosa (16) and in A. baumannii (22). When performing PCR amplifications of *piuA* from P. aeruginosa clinical isolates, we noticed the absence of a *piuA* signal in 54% of genotypically nonredundant isolates collected from intensive care unit patients (data not shown). We performed a homology search for potential orthologues of PiuA in the genome of LESB58, a strain that we previously showed lacks the *piuA* gene (16). The BLAST algorithm identified an open reading frame (ORF) of 766 amino acids in strains LESB58 (PALES_48941) and 39016 (PA39016_000870080), showing 60% amino acid identity with PiuA of PAO1 (753 amino acids). The highest sequence identity was observed in the N terminus (99% amino acid identity in the first 84 residues, including the signal sequence) and the putative substrate binding loops (NL1 to NL3) (see Fig. S1 in the supplemental material). We termed this PiuA orthologue PiuD. The *piuD* gene has a GC content of 59%, which is below the average of 66% for P. aeruginosa. To determine whether *piuD* would be present in strains from which *piuA* could not be amplified, we performed a multiplex PCR with *piuA* and *piuD* primer sets on the same set of genetically distinct clinical isolates tested above for *piuA*. The multiplex PCR confirmed our hypothesis, showing a PCR band either for *piuA* or for *piuD*, suggesting that both genes are mutually exclusive in P. aeruginosa genomes (see Fig. S2A). The *piuD* gene was found to be embedded in the same genomic context as *piuA* (16), since the gene products of *piuB* (PA4513) located downstream of *piuA* and those of the two genes *piuC* (PA4515) and *piuE* (PA4516), transcribed in an opposite direction, shared ≥98% amino acid identities with their homologues in LESB58 (Fig. S2B).

### Contribution of PiuD and PirA to the activity of siderophore-beta-lactam conjugates.

We compared the contributions of PiuA and PiuD with that of PirA, conserved in PAO1 and LESB58, to the activity of various siderophore-drug conjugates. To this end, we constructed deletion mutants in the *piuD* (PALES_48941) and *pirA* (PALES_43851) genes of LESB58. We tested the monobactam drugs BAL30072 (4) and MC-1 (5), conjugated to a hydroxypyridone siderophore, as well as the cephalosporin derivative cefiderocol, linked to a catechol siderophore (7) (Fig. 1). In the PAO1 background, both types of conjugates were strongly affected by the deletion of *piuA* (8- to 32-fold increase in MICs) but not by a *pirA* deletion. Surprisingly, the deletion of the *pirA* gene in the LESB58 background showed a stronger effect on the activities of the hydroxypyridone conjugates (8- to 16-fold increase in MICs) than on the catechol conjugate cefiderocol (2-fold increase in MICs). Conversely, the deletion of *piuD* increased cefiderocol MICs 32-fold, while MICs for MC-1 and BAL30072 increased by only 2- and 4-fold, respectively (Table 1). This could reflect the different expression levels of these receptors and/or the different affinities for the two types of siderophore-drug conjugates.

**TABLE 1:** Susceptibility of *piuD* and *pirA* deletion mutants of P. aeruginosa PAO1 and LESB58

Strain	MIC (mg/liter)^*a*^				
	BAL	MC-1	ATM	CFD	CAZ
PAO1	1	0.5	4	0.5	2
PAO1Δ*piuA*	8	8	4	8	2
PAO1Δ*pirA*	1	0.5	4	0.5	2
PAO1Δ*piuA*Δ*pirA*	16	16	4	16	2
LESB58	1	1	16	0.06	4
LESB58Δ*piuD*	4	2	16	2	4
LESB58Δ*pirA*	16	8	16	0.125	4
LESB58Δ*piuD*Δ*pirA*	32	32	16	4	4

a MICs were determined in MHB-Chelex. BAL, BAL30072; ATM, aztreonam; CFD, cefiderocol; CAZ, ceftazidime.

**FIG 2:**
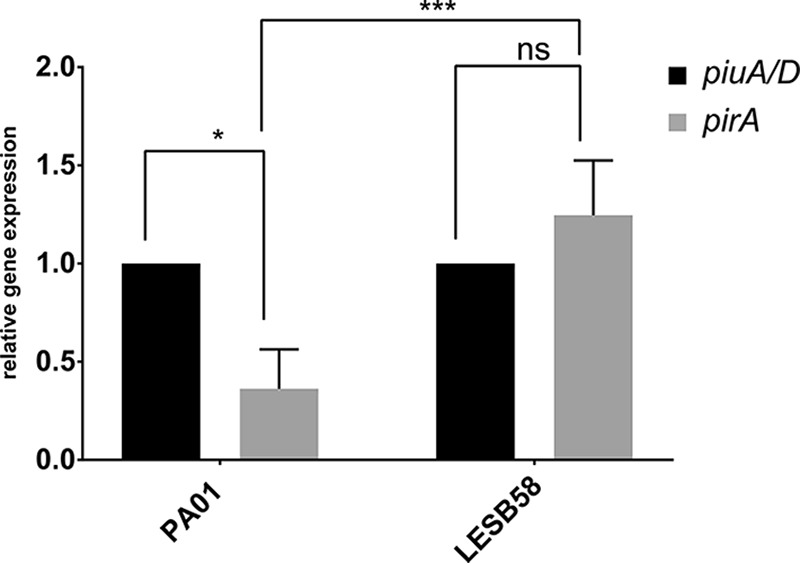
Expression analysis of *pirA* and *piuA* in PAO1 and *pirA* and *piuD* in LESB58. RNA was extracted from cells grown to late exponential phase in MHB. qPCR was performed using target-gene-specific primers. Expression of *pirA* is 3-fold lower than *piuA* in a PAO1 background, while *piuD* and *pirA* expression levels are comparable in strain LESB58. Values are the expression ratios of the target gene divided by the *rpsL* housekeeping gene. The expression of *piuA* and *piuD* was set to 1 (100%) in the respective strain. Values show the means from three independent experiments performed in duplicates. Error bars indicate standard deviations. *, *P* < 0.05 by Student *t* test; ***, *P* < 0.001 by analysis of variance (ANOVA); ns, not significant.

To address this question, we extracted RNA from PAO1 and LESB58 from late exponential-phase cells grown under the same conditions as for the MIC assays and measured by reverse transcription-quantitative PCR (qRT-PCR) the expression of *piuA* and *piuD* in comparison to that of *pirA*. As shown in Fig. 2, *pirA* was expressed 3-fold less than *piuA* in PAO1, while the relative expression levels between *pirA* and *piuD* were comparable in strain LESB58. The low basal expression level of *pirA* might therefore not be sufficient to contribute to siderophore-drug uptake in PAO1, as highlighted by the identical MIC values of the *pirA* mutant and the wild-type strain PAO1 (Table 1). In contrast, in LESB58, PirA seemed to transport preferentially the hydroxypyridones BAL30072 and MC-1, while cefiderocol uptake occurred mainly via PiuD. Since PirA amino acid sequences from PAO1 and LESB58 (PALES_43851) are 99% identical, this difference was not due to altered substrate affinities.

### Crystal structure of PiuD from P. aeruginosa.

**FIG 3:**
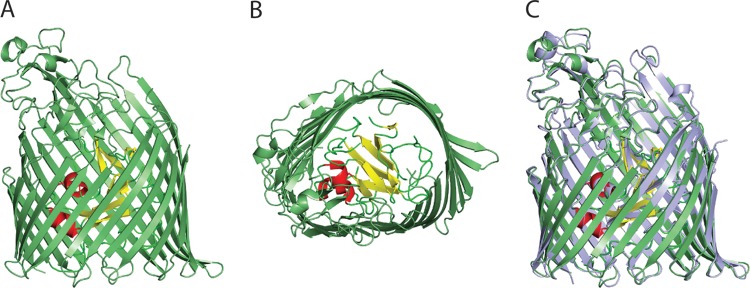
Crystal structure of PiuD from P. aeruginosa. Side (A) and extracellular (B) views of PiuD. The 22-stranded transmembrane β-barrel is colored in green. β-Sheets of the plug domain are colored in yellow, loops in green, and helices in red. (C) Structural comparison between PiuA (light blue) and PiuD (green).

We previously determined the crystal structure of PiuA from P. aeruginosa and its orthologue from A. baumannii (22). Here, we determined the structure of PiuD from strain 39016, which shows 99.6% amino acid identity with PiuD (PALES_48941) from LESB58. The obtained PiuD structure was similar to that of PiuA from PAO1. The crystallographic asymmetric unit has two monomers (denoted A and B). PiuD comprised two domains, a 22-stranded transmembrane β-barrel and an N-terminal plug domain (residues 27 to 156) folded inside the barrel (Fig. 3). The plug domain has two β-sheets and two α-helices, which together, occluded the central pore. As often occurs in the TBDR structures, some of the extracellular loops were not experimentally located in the PiuD structure. In the B monomer, these regions, namely, NL1 (83 and 84), NL3 (113 and 114), the loop 138 to 141 of the plug domain, L7 (504 to 530), L8 (564 to 572), and L9 (609 to 624), were presumed to be disordered. The closest structural relatives were PiuA of A. baumannii (root mean square deviation [RMSD] of 1.1 Å over 701 residues) and P. aeruginosa (1.1 Å over 656 residues) and the pyochelin receptor FptA from P. aeruginosa (1.8 Å over 655 residues) (23). As a consequence of the disorder, one side of the extracellular β-barrel was absent. A “belt” of outward facing hydrophobic residues (Trp 445, 486, 541, and 594, Phe 157, 180, 217, 350, 648, and 731, and Tyr 645 and 685), sits at the periplasmic end of the barrel, a characteristic of outer membrane proteins.

### Proteomic analysis under Fe chelation.

**FIG 4:**
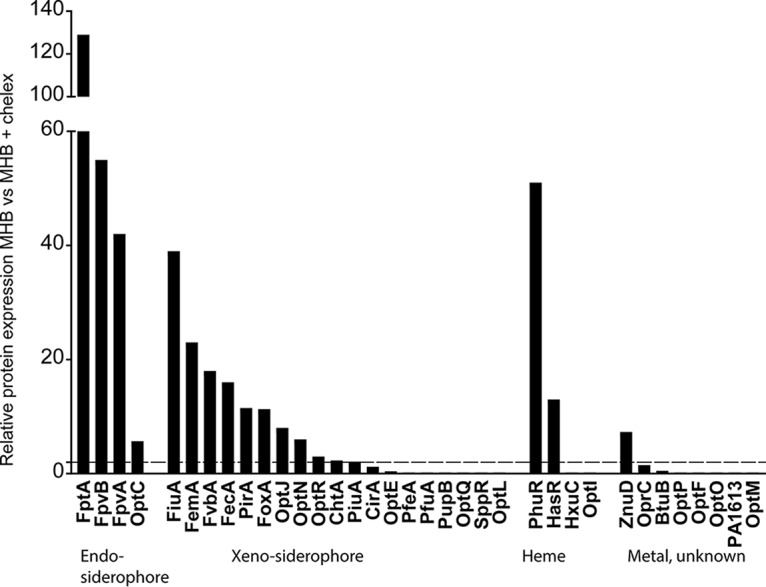
Proteomic analysis of TBDRs from P. aeruginosa PAO1. Protein expression levels were compared between cells grown for 20 h in MHB or Chelex-treated MHB. The dashed line indicates the 2-fold induction threshold level.

To identify further siderophore-drug transporters, we reasoned that under iron-limiting conditions, the expression of Fe-repressed TBDRs would be upregulated and could potentially contribute to the transport of siderophore-drug conjugates. Therefore, we performed a proteome analysis using PAO1 cells grown in Mueller-Hinton broth (MHB) and in MHB treated with Chelex, which complexes ferric iron but also divalent metal cations. The TBDRs for the endogenous siderophores pyochelin (FptA) and pyoverdin (FpvA and FpvB) showed the strongest induction in the Chelex-treated medium (40- to 130-fold increases) (Fig. 4). We also observed an induction of the heme receptors PhuR and HasR, as well as of the Zn transporter ZnuD. The expression of the known siderophore-drug transporters PiuA and PirA increased 2- and 10-fold, respectively, upon iron chelation. Among the TBDRs expected or reported to transport xenosiderophores, 11 showed a >2-fold increase in expression. CirA was below the 2-fold induction threshold, and six xenosiderophore receptors were not expressed or were expressed below the detection limit.

### Constitutive expression of TonB-dependent receptors in PAO1.

To assess the possible involvement of these receptors in siderophore-drug uptake, we cloned 26 of the 34 TBDR genes from PAO1 (see Table S1), including the Chelex-induced TBDRs (FiuA, FemA, FoxA, OptJ, OptN, ChtA, and CirA) and those that were undetectable. We cloned the corresponding genes into a vector harboring a constitutively expressed promoter in P. aeruginosa and transferred the resulting plasmids into strain PAO1. We excluded the heme/hemophore transporters (PhuR, HasR, HxuC, and OptI), the cobalamin transporter BtuB (PA1271), and the citrate receptor FecA. For comparison, we included the previously identified receptor genes *piuA* and *pirA*, as well as the newly identified *piuA* orthologue *piuD*. The susceptibility data clearly showed that six TBDRs, namely, PiuD, OptJ, FemA, OptE, PfuA, and FptA, increased by at least 4-fold the susceptibility of PAO1 to the three siderophore-drug conjugates tested (shown in bold in Table 2). The strain harboring pPA0151 showed a 4-fold increase in susceptibility only for the dihydroxypyridone-containing drugs BAL30072 and MC-1, and the strain harboring ChtA only showed increased susceptibility for the catechol-based cefiderocol. Surprisingly, the overexpression of PfuA, which was undetectable by proteome analysis, produced the largest increase in susceptibility (>32-fold for BAL30072). With the exception of a 4-fold-decreased MIC for ceftazidime (pPA0151), we observed no significant changes in MICs for the nonsiderophore drugs aztreonam and ceftazidime. Since OptJ was induced to a similar level as PirA under Chelex treatment (Fig. 4), we constructed deletions in *optJ* in PAO1 and its *piuA* and *pirA* deletion mutants. As for a *pirA* deletion in PAO1, *optJ* deletion had no effect on siderophore-drug conjugate MICs (see Table S4). However, a consistent 2-fold increase in BAL30072 MICs in a *piuA* deletion background suggests a minor contribution of OptJ under uninduced conditions in a PAO1 background.

**TABLE 2:** Effect of overexpression of TonB-dependent receptors on P. aeruginosa susceptibilities to three siderophore-drug conjugates

Strain or plasmid^*a*^	MIC (mg/liter)^*b*^				
	BAL	MC-1	ATM	CFD	CAZ
PAO1	1	0.25	4	0.5	1
pIApX2 (vector)	1	0.25	4	0.5	1
ppiuA1.1	0.06–0.125	0.06	4	0.03–0.06	1
ppirA1.1	0.06–0.125	0.06	4	0.03–0.06	1
**ppiuD**	**0.06–0.125**	**0.03**	4	**0.03**	1
**poptJ** (PA0434)	**0.06–0.125**	**0.03**	4	**0.03–0.06**	1
**pfemA** (PA1910)	**0.125**	**0.06**	2	**0.06**	1
**poptE** (PA2911)	**0.25**	**0.06**	2	**0.125**	1
**ppfuA** (PA1322)	**0.03**	**0.03**	2	**0.03**	1
**pfptA**	**0.125**	**0.06**	2	**0.125**	0.5
pPA0151	**0.25**	**0.06**	2	0.25	**0.25**
pchtA (PA4675)	0.5	0.125	2	**0.125**	ND^*c*^
pfiuA (PA0470)	0.5	ND	4	0.25	1
pfoxA (PA2466)	1	0.125	2	0.25	1
ppfeA (PA2688)	1	0.25	4	0.5	ND
pcirA (PA1922)	1	0.25	4	0.25–0.5	1
poptN (PA1365)	1	0.25	4	0.5	ND
poptF (PA2590)	1	ND	4	0.5	0.5
poptQ (PA2289)	1	ND	2	0.25	0.5
poptR (PA3268)	1	0.25	2	0.5	1
pznuD (PA0781)	1	ND	2	ND	ND
optO (PA2335)	0.5	0.25	2	0.5	1
poptP (PA0192)	1	0.25	2	0.5	1
poptL (PA2089)	1	0.25	4	0.5	1
poptC (PA4837)	1	0.125	2	0.5	1
pPA1613	1	0.125	4	0.5	1
poptM (PA2070)	1	0.25	4	0.5	1
psppR (PA2057)	0.5	0.25	4	0.5	2
pfvbA (PA4156)	0.5	0.125	4	0.5	0.5
pfpvA	2	0.5	8	0.5	1
pfpvB	1	0.25	4	1	1

a Plasmids in boldface font conferred a ≥4-fold increase in susceptibility to all siderophore-drug conjugates compared to the vector control.

^a^BAL, BAL30072; ATM, aztreonam; CFD, cefiderocol; CAZ, ceftazidime.

c ND, not done.

To assess whether the observed changes in susceptibility could result from indirect effects on the expression level of the main siderophore-drug transporter PiuA, we introduced the relevant constructs in a PAO1Δ*piuA* deletion mutant and tested the drug susceptibilities. PiuA, PirA, and PiuD expression decreased the MICs of all three siderophore-drug conjugates by 8- to ≥32-fold (Table 3). Interestingly, PirA overexpression produced only a 4- to 8-fold MIC decrease for cefiderocol compared to that of the vector control, while PiuD expression resulted in a ≥32-fold MIC decrease (Table 3). This finding is in agreement with the susceptibilities of *pirA* and *piuD* mutants in strain LESB58 (Table 1), which suggested preferential transport of cefiderocol via PiuD. The overexpression of PfuA showed MIC changes exceeding those conferred by PiuA and PirA, suggesting efficient siderophore-drug transport independent of PiuA. Similar results were obtained in a *piuA-pirA* double mutant (data not shown). On the other hand, FptA and OptE expression produced 2- to 8-fold decreases for MC-1 and cefiderocol, and OptJ produced a decrease only for MC-1. Finally, FemA and PA0151 expression showed no significant MIC changes in a *piuA* deletion mutant, suggesting an indirect effect on PiuA expression, when overexpressed in a PAO1 wild-type strain.

**TABLE 3:** Effect of overexpression of TonB-dependent receptors on siderophore-drug conjugates activities in a *piuA* deletion mutant of P. aeruginosa

Strain or plasmid	MIC (mg/liter)^*a*^				
	BAL	MC-1	ATM	CFD	CAZ
PAO1Δ*piuA*	8	2	8	8	2
pIApX2 (vector)	8	4	4	8	2
ppiuA	**0.25**	**0.03**	4	**0.06**	2
ppirA	**0.25**	**0.06**	4	**1–2**	2
ppiuD	**0.125**	**0.03**	8	**0.03–0.125**	2
ppfuA	**0.03**	**0.03**	4	**0.03**	2
pfptA	4	**0.5**	2	**0.25**	2
poptE	4	**0.5**	4	**2**	2
poptJ	4	**1**	4	4–8	2
pfemA	8	2	4	8	2
pPA0151	8	4	2	8	1

a MIC changes of ≥4-fold compared to the vector control are shown in boldface font. BAL, BAL30072; ATM, aztreonam; CFD, cefiderocol; CAZ, ceftazidime.

To further evaluate if additional TBDRs would be involved in the uptake of siderophore-drug conjugates, we determined the susceptibilities under iron-limited growth conditions in MHB Chelex and in a minimal Casamino Acids medium. We observed 2-fold decreases in MICs of BAL30072 and MC-1 in PAO1 and the *pirA* mutant, and a 4- to 8-fold drop in the *piuA* mutant backgrounds. The increase in susceptibility was even more pronounced for cefiderocol (8- to 64-fold decreases) for the strains tested. MICs for the nonsiderophore drugs aztreonam and ceftazidime were not affected (see Table S5). The MICs were comparable or even lower than those obtained by the overexpression of the individual receptors from the plasmids in the *piuA* mutant background (Table 3), suggesting a simultaneous expression of several TBDRs besides PiuA and PirA for the uptake of siderophore-drug conjugates in P. aeruginosa under iron-limited conditions.

## DISCUSSION

The Trojan horse strategy has recently gained renewed interest, as illustrated by the development of novel siderophore-beta-lactam conjugates by pharmaceutical companies (4, 12) and academic research groups (9, 24). These differ in the beta-lactam scaffolds (penicillins, monobactams, and cephems) as well as the attached siderophore moieties (mono-, tris-catechols and mixed catechol-hydroxamates). Initial investigations have identified two TBDR proteins, PiuA and PirA, in P. aeruginosa (5, 16) and their orthologues in A. baumannii (22). These are the main transporters for BAL30072 and MC-1. While the deletion of these TBDRs affected the activity of these compounds under standard MIC determination conditions, it remained unclear whether additional TBDRs expressed under iron deficiency or upon substrate-induced expression can contribute to drug susceptibility.

We have addressed these questions by screening for orthologues of PiuA in clinical strains and by overexpressing 28 of the 34 TBDRs from P. aeruginosa PAO1, thereby mimicking induction under specific physiological conditions or by natural substrates. An *in silico* screen identified PiuD in LESB58 and other clinical isolates as a homologue of PiuA, sharing 60% amino acid identity. The *piuD* gene was located in the same genetic environment as *piuA*, including the conserved intergenic promoter region (see Fig. S2 in the supplemental material). The lower GC content of the *piuD* gene (59% compared to 66% for PAO1) suggests an acquisition by horizontal gene transfer. The natural substrates of PiuA and PiuD are unknown, but the presence of conserved genes within the *piu* locus, including the oxidoreductase genes *piuC* and *piuB* and the ORF PA4516 (*piuE*), suggests that the metabolic fates of the natural substrates of these receptors are similar.

The amino acid similarity between PiuA and PiuD (Fig. S1) results in very similar crystal structures (Fig. 3). Like PiuA, PiuD also has a distinctive cluster of aromatic and positively charged residues located inside the pore at the extracellular face (see Fig. S3). This cluster is formed by Trp residues 311 and 327, Tyr 309, 710, and 714, Phe 94 (from the plug domain), His 713, and Arg 329 and 333 (Fig. S3). In PiuA, Trp 239, Tyr 307, 325, and 697, Phe 94, His 700, Lys 329, and Arg 331 form a cluster in the same position (Fig. S3). In the pyoverdin (FpvA) and pyochelin (FptA) receptors, this cluster is directly involved in the recognition of siderophores (23, 25). So far, there is no cocrystal structure available for a TBDR with its siderophore-drug conjugate, and only two cocomplexes between natural siderophores and their corresponding receptors have been solved (26–28). However, several binding and mutation studies regarding siderophore receptors and their cognate substrates have been reported (29–31), and their results are compatible with biphasic binding kinetics involving an initial binding in the loop extremities and a secondary binding at a site deeper inside the barrel, leading eventually to substrate translocation.

Our proteomic analysis revealed that divalent metal cation chelation induced the expression of 18 of the 34 TBDRs in PAO1. These include receptors for the endogenous siderophores pyoverdin (FpvA and FpvB), pyochelin (FptA), and nicotianamine (OptC) (32), as well as the heme (PhuR and HasR) and zinc (ZnuD) transporters. The other induced receptors could transport xenosiderophores that P. aeruginosa may encounter in the environment or during polymicrobial infections. A subset of these likely requires the cognate siderophore as an inducer. One example is PfeA from PAO1, sensing the presence of the exogenous siderophore enterobactin from Escherichia coli via the two-component system PfeR-PfeS (33). Similarly, the siderophore mycobactin from Mycobacterium smegmatis induces by 30-fold the expression of FemA in P. aeruginosa (19). Strikingly, the overexpression of PfuA resulted in the largest increase in susceptibility to all three siderophore-drug conjugates tested. The closest homologues of PfuA turned out to be PiuA in PAO1 and PiuD in LESB58, both sharing a 39% amino acid identity (57% similarity). The natural substrate of PfuA is unknown. A Fur binding site precedes the *pfuA* gene (34), suggesting iron repression; however, additional regulators and the presence of the substrate are likely required for induction of this TBDR in PAO1. Its closest orthologue in E. coli is Fiu, a TBDR also involved in the transport of BAL30072 (our unpublished data). Other receptors, undetectable by mass spectrometry (MS) analysis, may respond to other organic compounds or metal ions. Importantly, we identified the pyochelin receptor FptA as a candidate for the uptake of siderophore-drug conjugates. This receptor is the most highly induced receptor under iron limitation, as highlighted by our proteome analysis. FptA is also strongly expressed in lung and blood samples from mice and rats infected with P. aeruginosa and in human urine and respiratory samples (D. Bumann, unpublished results). The identification of the natural substrates of xenosiderophore receptors, as for instance PfuA, should provide an elegant way to induce specifically the expression of a receptor for the uptake of siderophore-drug conjugates. It also remains to be determined if siderophore-drug conjugates can act as inducers of their own transport, although this would require conjugate analogues deprived of antibiotic activity. The increased susceptibility to all three siderophore-drug conjugates under iron-limited conditions supports our findings on the plasmid-mediated expression of the individual TBDRs.

In summary, we have provided evidence for an overlapping subset of TBDRs in P. aeruginosa able to transport three different siderophore-drug conjugates, presenting two different types of iron-complexing substituents and on the basis of two classes of beta-lactams. The redundancy of TBDR recognition profiles should be an advantage during therapeutic treatments, since it should limit the risk of resistance emergence to these novel drug conjugates.

## MATERIALS AND METHODS

### Bacterial strains and growth conditions.

The strains and plasmids used in this study are listed in Table S1 in the supplemental material. E. coli and P. aeruginosa were grown in lysogeny broth (LB) at 37°C with shaking (250 rpm). E. coli DH10B was used as the cloning host and E. coli SM10 as the donor for biparental matings. Gentamicin (15 μg/ml for E. coli and 50 μg/ml for P. aeruginosa) or carbenicillin (200 μg/ml) was added for plasmid -carrying strains. MICs were determined in Mueller-Hinton broth (MHB) according to CLSI guidelines (35) and were repeated at least on three different occasions. Cation-depleted MHB was prepared by dissolving 11 g of Chelex (C7901; Sigma-Aldrich) in 100 ml MHB. After stirring for 6 h, the suspension was filtered and the filtrate autoclaved at 115°C for 15 min. The Chelex-treated MHB was supplemented with 2 mM MgSO_4_ and 0.2 mM CaCl_2_ (final concentrations). The M9 Casamino Acids medium contained 1× M9 salts, supplemented with 0.5% Casamino Acids (filter sterilized), and 2 mM MgSO_4_.

### PCR amplifications and DNA modifications.

PCR primers are listed in Table S2. All primer sequences were based on the sequences from the pseudomonas.com website (36). For screening PCRs, bacterial cells were boiled at 95°C for 5 min and subsequently pelleted at 13,000 rpm for 1 min. Phusion DNA polymerase (Thermo Scientific) was used for high-fidelity PCRs (supplemented with 5% dimethyl sulfoxide [DMSO]). Restriction digestions were performed according to the manufacturer's instructions at the appropriate temperature. All ligation reactions were carried out at room temperature using T4 DNA ligase (Promega). DNA preparations were performed using the GeneJET PCR purification or the GeneJET gel extraction kit (Thermo Scientific).

### Construction of knockout mutants.

The generation of unmarked knockout mutants was based on the protocol described by Hoang et al. (37). Briefly, DNA fragments of 500 to 700 bp were PCR-amplified using primer pairs A1/A2 and B1/B2, respectively. For the deletion of *pirA* in strain LESB58, the up- and downstream regions flanking the gene were PCR amplified. For the knockout of *piuD* in strain LESB58, the amplified fragments were located in the 5′ and 3′ regions of the genes. After amplification, the obtained A and B fragments were gel purified, and approximately 40 ng of each fragment was used in a PCR fusion amplification with primers A1 and B2, which share an 18-bp homologous region. The resulting fusion products were gel purified and further cloned into the suicide vectors pEX18Gm via HindIII/EcoRI restriction sites (*pirA*) and pEX18Gm via SalI/EcoRI (*piuD*). The cloned knockout fragments were verified by sequencing. The replacement vectors were mobilized into P. aeruginosa via biparental conjugation, and the generation of the unmarked mutants was carried out as previously described (38). The defined gene knockouts were verified by PCR amplification using the external primers and subsequent Sanger sequencing.

### Construction of expression plasmids.

The coding regions, including at least 50 nucleotides (nt) upstream of the ATG initiation codon and 20 nt downstream of the STOP codon, were amplified by PCR from genomic DNA of P. aeruginosa 39016 (*piuD*) or PAO1. The *piuD* coding region was amplified with primers piuD-Xba and piuD-Hind and cloned as a 2,526-bp XbaI-HindIII DNA fragment into the expression vector pIApX2, yielding plasmid ppiuD. All other constructs were prepared in a similar way using the primers shown in Table S2. The Q5 high-fidelity DNA polymerase (NEB) was used for all amplifications. PCR conditions were as follows: denaturation at 98°C for 2 min, followed by 27 cycles of 98°C for 20 s, 57°C for 30 s, and 72°C for 2 min, and a final extension at 72°C for 4 min. The plasmids were transferred into P. aeruginosa by electroporation, and cells were spread on LB agar supplemented with carbenicillin at 200 mg/liter. All constructs were verified by Sanger sequencing.

### Quantitative real-time PCR.

Overnight cultures of strains grown in LB were diluted and inoculated into fresh MHB and grown in microtiter plates (200 μl/well) until reaching late exponential phase. Three wells were combined to form one sample. RNA was extracted using the RNeasy kit (Qiagen, Germany), according to the manufacturer's protocol. Residual genomic DNA was removed by treatment with RNase-free DNase (Promega). One microgram of RNA was reverse transcribed using ImProm-II reverse transcriptase (Promega). Gene-specific primers were used for PCRs using the Rotor-Gene SYBR green PCR kit (Qiagen). qPCRs were performed in a Rotor-Gene 3000 (Corbett Research, Australia) using the following conditions: 2 min at 95°C, followed by 35 cycles of 20 s at 95°C, 30 s at 60°C, and 30 s at 72°C, followed by a final extension at 72°C for 3 min. The ribosomal *rpsL* gene was used as a housekeeping reference gene (39).

### Cloning, overexpression, and purification of PiuD from P. aeruginosa.

The signal peptide of the proteins was predicted with Signal P4.0 (40) and excluded from cloning. The coding sequence of the mature protein was amplified from the genomic P. aeruginosa strain 39016 using KOD DNA polymerase (Novagen) and the primers piuD-39016-F and piuD-39016-R. The PCR product was digested by BspHI and XhoI restriction enzymes and cloned into the pTAMACHis6 vector using restriction sites NcoI and XhoI. The construct results in an expressed protein with an N-terminal TamA signal sequence for the outer membrane localization and a noncleavable C-terminal His_6_ tag. The pTAMACHis6 expression vector was obtained by replacing the PelB signal peptide of pEPELBCHIS (courtesy of Huanting Liu, University St Andrews) with the TamA signal peptide (41). PiuD was overexpressed in E. coli C43(DE3) cells. The expression and purification steps were as described for PiuA (22). The fractions were pooled and loaded on a Superdex S200 gel filtration column (GE Healthcare) equilibrated with 10 mM Tris (pH 8), 150 mM NaCl, and 0.45% (vol/vol) tetraethylene glycol monooctyl ether (C8E4). Protein fractions were pooled and concentrated to 10 mg/ml.

### Crystallization and structure determination.

Crystals of PiuD appeared at 20°C after a few days by mixing 2 μl of protein solution (10 mg/ml) with 1 μl of reservoir solution containing 14% poly(ethylene glycol) methyl ether (PEG MME 5000) and 0.1 M bicine (pH 9). Crystals were frozen with the same solution containing 35% PEG MME 5000. The data were collected at ID23-1 at the ESRF. The data were processed with GrenADES (42–46). The structure of PiuD was solved by molecular replacement using P. aeruginosa PiuA coordinates (PDB code 5FOK) as a model, with the program PHASER (47). The models were adjusted with Coot (48), and the refinement was carried out using REFMAC in the CCP4 program suite with TLS parameters (49). The quality of all structures was checked with MolProbity (50). The figures were drawn using PyMOL (version 1.8; Schrödinger, LLC). The final refinement statistics are given in Table S3.

### Proteomics analysis.

Sample preparation and MS analysis were performed as described previously (33). Briefly, P. aeruginosa was grown in MHB or MHB treated with Chelex (Sigma-Aldrich, Switzerland) under standard MIC determination conditions in microtiter plates without shaking at 37°C for 18 h. The cells from three wells were combined to yield sufficient material for proteome analysis. Three replicate samples were lysed, and the proteins were reduced with 5 mM Tris (2-carboxyethyl) phosphine hydrochloride and alkylated with iodoacetamide. The samples were diluted before digestion with trypsin at 37°C overnight. The peptides were desalted on a C_18_ reversed-phase column and dried under vacuum. One microgram of peptide was injected into a liquid chromatography-mass spectrometer (LTQ-Orbitrap Elite). The peptides were separated using an EASY nLC-1000 system (Thermo Fisher scientific) using a C_18_ high-performance liquid chromatography (LC) column. Tandem mass spectrometry data were exported from Progenesis LC-MS and searched against a protein decoy database of P. aeruginosa.

### Statistics.

Data were analyzed and plotted using GraphPad Prism (ver 7.02).

### Accession number(s).

Atomic coordinates and structure factors for PiuD have been deposited in the Protein Data Bank (accession no. 5NEC).

## Supplementary Material

Supplemental material
