# Supplementary material for: TonB-Dependent Receptor Repertoire of Pseudomonas aeruginosa for Uptake of Siderophore-Drug Conjugates
Source: Antimicrob Agents Chemother. 2018 May 25;62(6):e00097-18. doi: 10.1128/AAC.00097-18 (PMC5971595; doi:10.1128/AAC.00097-18)
Supplement: Supplemental material [file supp_62_6_e00097-18__index.html]

Supplemental material 

# TonB-Dependent Receptor Repertoire of Pseudomonas aeruginosa for Uptake of Siderophore-Drug Conjugates

## Supplemental material

- Supplemental file 1 -

  Fig. S1 to S3 and Tables S1 to S5

  PDF, 1.4M
