# Supplementary material for: TonB-Dependent Receptor Repertoire of Pseudomonas aeruginosa for Uptake of Siderophore-Drug Conjugates
Source: Antimicrob Agents Chemother. 2018 May 25;62(6):e00097-18. doi: 10.1128/AAC.00097-18 (PMC5971595; doi:10.1128/AAC.00097-18)
Supplement: Supplemental material [file AAC.00097-18_zac006187173s1.pdf]

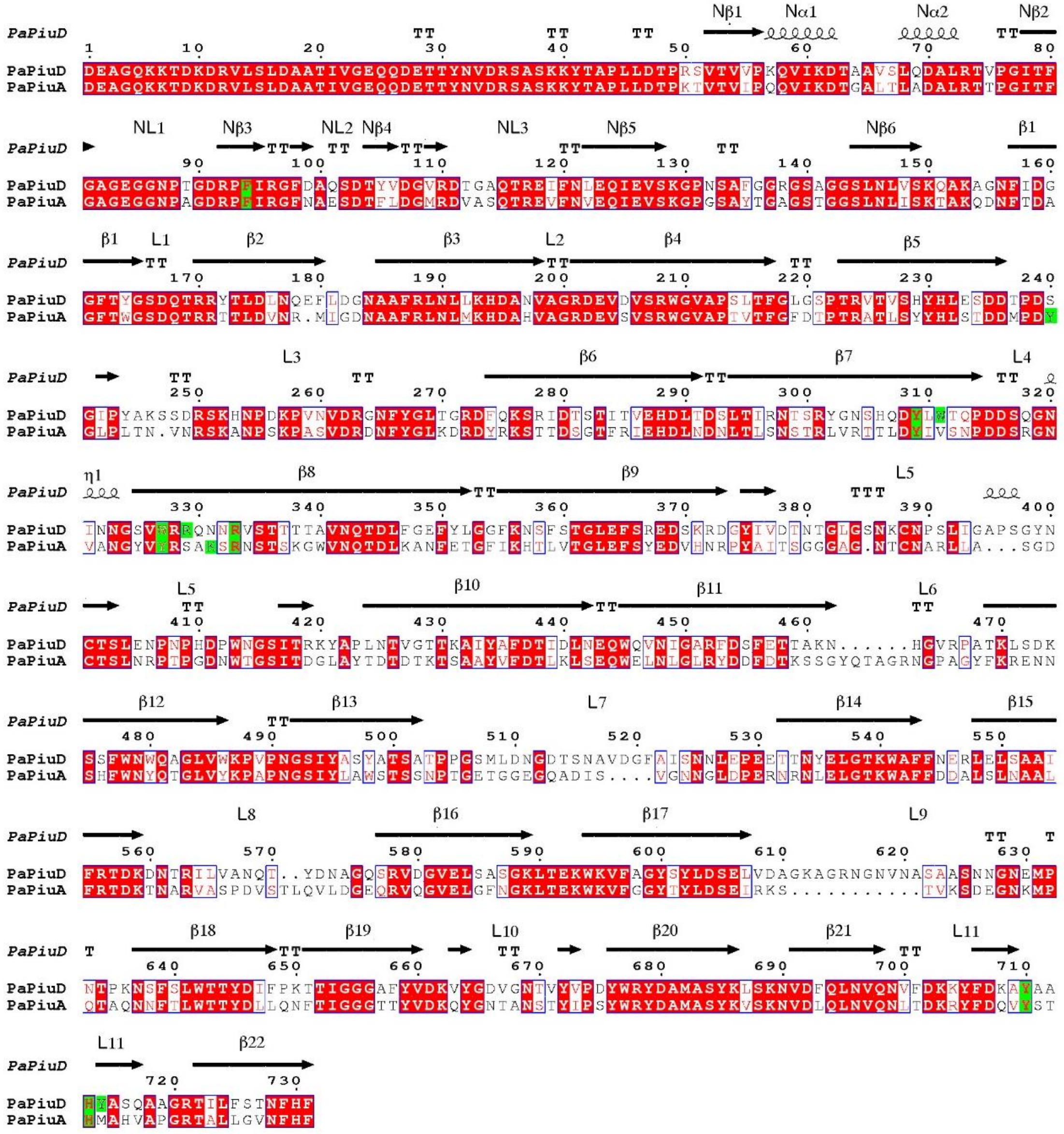

FIG. S1. Structure-based sequence alignment of PiuD and PiuA. Secondary structure elements of PiuD are illustrated above the alignment (numbering with respect to PiuD without the signal sequence). Residues forming the cluster of aromatic and positively charged residues are highlighted in green (see also Fig. S3). The alignment was performed with PROMALS3D (4) and drawn using ESPript (5).

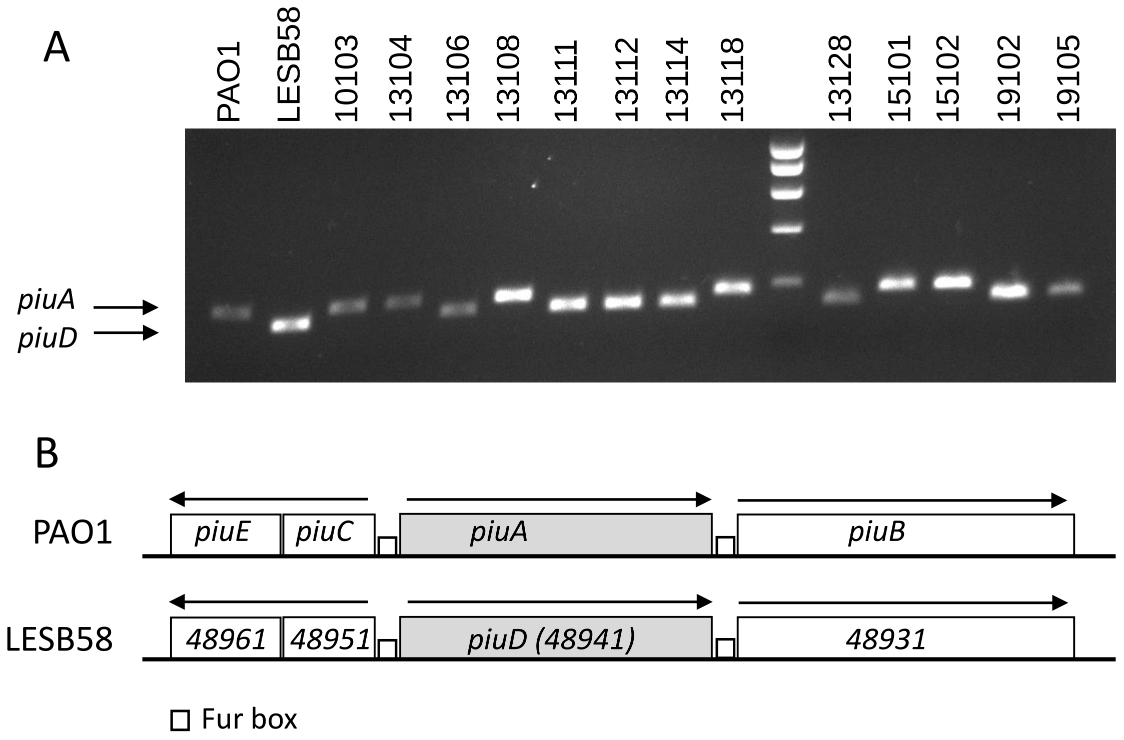

FIG. S2. Distribution of *piuA/piuD* genes in clinical isolates. (A) Multiplex PCR using *piuA* specific (PAO1) and *piuD*-specific (LESB58) primers was performed on genomic DNA from 13 genetically distinct isolates previously collected from ICU patients. Multiplex PCR shows a signal for either of the two genes in all isolates tested. (B) Strains carry either *piuA* or *piuD* genes located within a conserved genetic locus on the *P. aeruginosa* chromosome. Only *piuA* and *piuD* sequences (shaded in grey) differ between the two loci. Fur-boxes located upstream of *piuA/piuD* and *piuB* genes are conserved and indicated by a square. Arrows indicate putative operon structure.

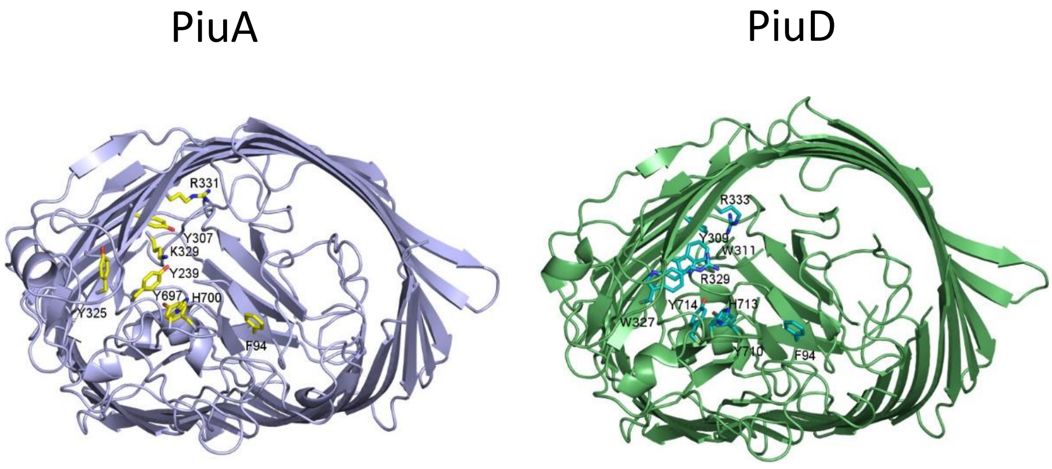

Fig. S3. Cluster of aromatic and charged residues in PiuA (light blue) and PiuD (green) *of P. aeruginosa*. Residues of the cluster are shown as sticks in yellow for PiuA and aquamarine for PiuD.

Table S1 Bacterial strains and plasmids

Strains/plasmids	Relevant characteristics	Reference, source
P. aeruginosa		
PAO1	PAO1, *mexT* non-functional	Laboratory collection
Δ*piuA*	unmarked deletion of *pirA* gene	(1)
Δ*pirA*	unmarked deletion of *pirA* gene	(2)
Δ*piuA*Δ*pirA*	unmarked deletion of *pirA, piuA* genes	(2)
Δ*optJ*	unmarked deletion of *optJ* gene	This study
Δ*piuA*Δ*pirA*Δ*optJ*	unmarked deletion of *optJ, pirA, piuA* genes	This study
LESB58		
Δ*piuD*	unmarked deletion of *piuD* gene	This study
Δ*pirA*	unmarked deletion of *pirA* gene	This study
Δ*piuD*Δ*pirA*	unmarked deletion of *pirA, piuD* genes	This study
E. coli		
ST18	*pro thi hsdR*$^{+}$ Tmp^{r} Sm^{r}*;* chromosome::RP4-2 Tc::Mu-Kan::Tn7/λpirΔ*hemA*	(3)
**Plasmids**		
pEXG2	gene replacement vector, Gm-R	
pIApX2	Broad-host range expression vector, Ap-R	I. Attree (Grenoble, France)
ppiuA1.1	*piuA* from PAO1 cloned into pIApX2, Ap-R	(2)
ppirA1.1	*pirA* from PAO1 cloned into pIApX2, Ap-R	(2)
ppiuD	*piuD* from LESB58 cloned into pIApX2, Ap-R	This study
poptJ	*optJ* (PA0434) from PAO1 cloned into pIApX2, Ap-R	This study

pfemA	*femA* (PA1910) from PAO1 cloned into pIApX2, Ap-R	This study
poptE	*optE* (PA2911) from PAO1 cloned into pIApX2, Ap-R	This study
ppfuA	*pfuA* (PA1322) from PAO1 cloned into pIApX2, Ap-R	This study
pfiuA	*fiuA* (PA0470) from PAO1 cloned into pIApX2, Ap-R	This study
pchtA	*chtA* (PA4675) from PAO1 cloned into pIApX2, Ap-R	This study
ppfeA	*pfeA* (PA2688) from PAO1 cloned into pIApX2, Ap-R	This study
pcirA	*cirA* (PA1922) from PAO1 cloned into pIApX2, Ap-R	This study
poptN	*optN* (PA1365) from PAO1 cloned into pIApX2, Ap-R	This study
poptF	*optF* (PA2590) from PAO1 cloned into pIApX2, Ap-R	This study
poptQ	*optQ* (PA2289) from PAO1 cloned into pIApX2, Ap-R	This study
pfoxA	*foxA* (PA2466) from PAO1 cloned into pIApX2, Ap-R	This study

pfpvA	*fpvA* (PA2389) from PAO1 cloned into pEX1.8, Ap-R	This study
pfpvB	*fpvB* (PA4168) from PAO1 cloned into pEX1.8, Ap-R	This study
pfptA	*fptA* (PA4221) from PAO1 cloned into pIApX2, Ap-R	This study
poptO	*optO* (PA2335) from PAO1 cloned into pIApX2, Ap-R	This study
poptP	*optP* (PA0192) from PAO1 cloned into pIApX2, Ap-R	This study
poptL	*optL* (PA2089) from PAO1 cloned into pIApX2, Ap-R	This study
pPA1613	PA1613 from PAO1 cloned into pIApX2, Ap-R	This study
poptR	*optR* (PA3268) from PAO1 cloned into pIApX2, Ap-R	This study
pfvbA	*fvbA* (PA4156) from PAO1 cloned into pIApX2, Ap-R	This study
ppupB	*pupB* (PA0151) from PAO1 cloned into pIApX2, Ap-R	This study
psppR	*sppR* (PA2057) from PAO1 cloned into pIApX2, Ap-R	This study

poptM	*optM* (PA2070) from PAO cloned into pIApX2, Ap-R	This study
pznuD	*znuD* (PA0781) from PAO1 cloned into pIApX2, Ap-R	This study
pEX18Gm-pirA-ko	contains a 1.4-kb fusion fragment of the up- and downstream region of *pirA from* LESB58, Gm-R	This study
pEX18Gm-piuD-ko	1.1-kb fusion fragment of the 5' and 3' region of *piuD* from LESB58, Gm-R	This study
pEXG2-optJ-ko	contains a 1.4-kb fusion fragment of the up- and downstream region of *optJ*, Gm-R	This study

[a] Ap, ampicillin; Gm, gentamicin; Km, kanamycin; Sm, streptomycin, Tc, tetracycline; Tmp, trimethoprim.

TABLE S2. Primers used in this study

Primer	Sequence (5' - 3')	Characteristic
piuA1	TACTTCAAGCGCGAGAACAACA	qPCR
piuA2	CCAGTTCGAGGTTACGGTTACG	qPCR
pirA1	AACAAGACCGATTCGGACGAT	qPCR
pirA2	GTGCCGTTGTTGTTCTGGGTA	qPCR
piuD-Xba	ACACTCTAGATTGGCCATGTAAAACCGCC	cloning
piuD-Hind	ACACAAGCTTGAAAGCCCCATCCCGTAGAG	cloning
piuD1	CGATCCATGGAATGGCAGTATT	qPCR
piuD2	GGTCTTACACCGTGGTTCTTCG	qPCR
piuD-39016-F	AGGGGCGTCATGAACGAGGCCGGGCAGAAAAAG	Purification of PiuD
piuD-39016-R	AATTTCTCGAGGAAGTGGAAGTTGGTGCTGAACAGT	Purification of PiuD
OptJ-Bam	ACACGGATCCCATTTGCATCCGTCGCCCG	Expression of OptJ
OptJ-Hind	ACACAAGCTTGAGACGAACCAGAGCACCATCA	Expression of OptJ
OptQ-Hind	ACACAAGCTTGTCGCGGAAAATTCTCGTTG	Expression of OptQ
OptQ-Xba	ACACTCTAGAAAGCCCCTTCGATACCTTG	Expression of OptQ
FptA-Bam	ACACGGATCCCCGGAGACGATTTCTTCATAA	Expression of FptA
FptA-Hind	ACACAAGCTTAGCCCGCGAGTCAACAGATAG	Expression of FptA
OptF-Bam	ACACGGATCCAATTCATATTGCCCGACGCTG	Expression of OptF
OptF-Nsi	ACACATGCATGTCGCGGAAAATTCTCGTTG	Expression of OptF
FiuA-Xba	ACACTCTAGAGGAGGCATTGAGACTCGTTT	Expression of FiuA

FiuA-Hind	ACACAAGCTTGAAGGTACGTTCTGCGGTAGG	Expression of FiuA
FoxA-Xba	ACACTCTAGACGGCTTTACCGATTGAACAC	Expression of FoxA
FoxA-Hind	ACACAAGCTTGGTAGCCAGACCGACATAGC	Expression of FoxA
FemA-Xba	ACACTCTAGACGACCTGAGACCTTTTTCCTT	Expression of FemA
FemA-Hind	ACACAAGCTTAGATCAGGCTGGTCCATTTGT	Expression of FemA
FhuA-Xba	ACACTCTAGAGACTCTGCCGTTGGTTATCAG	Expression of FhuA
FhuA-Hind	ACACAAGCTTCGCAGTGAAAAAGTTCAGAGG	Expression of FhuA
OptE-Xba	ACACTCTAGACCCGGTCATGAGATGGAAC	Expression of OptE
OptE-Hind	ACACAAGCTTGAAGGGACGACATGGCTACT	Expression of OptE
ChtA-Xba	ACACTCTAGACATGCCTCATTGAAACAGT	Expression of ChtA
ChtA-Hind	ACACAAGCTTGCTCTTTCCCACGAGTTCC	Expression of ChtA
CirA-Xba	ACACTCTAGACTCGCCTCGATGAAGAAGAAAC	Expression of CirA
CirA-Hind	ACACAAGCTTGCCGCAGAAGAGTAGCGAAC	Expression of CirA
OptN-Bam	ACACGGATCCGTCGAGATCCAACGCAAATC	Expression of OptN
OptN-Xba	ACACTCTAGAGCACACTGAGCGCAATTGG	Expression of OptN
PfeA-Bam	ACACGGATCCTCTTGCATTTCGCTGCATTG	Expression of PfeA
PfeA-Hind	ACACAAGCTTCGACGAGCAGGGAGGTTCTC	Expression of PfeA
PA0781-Bam	ACACGGATCCGGGTCTACCGACACAAAAAGA	Expression of ZnuD
PA0781-Hind	ACACAAGCTTGGGAGTGGTGTAGGACGGATAA	Expression of ZnuD
PA4156-F-Bam	ACACGGATCCGACGGGCGTAGGAGATTTCA	Expression of FvbA
PA4156-R-Hind	ACACAAGCTTGAAGATGCCTTCGATGTTGCT	Expression of FvbA

PA2089-Bam-F	ACACGGATCCTGAAAATCGGTACGGGCAAT	Expression of OptL
PA2089-Hind-R	ACACAAGCTTAGTTCGATGCTCATGGTTCGT	Expression of OptL
PA2070-Bam-F	ACACGGATCCTCTAAGCCGTCCCTGGAAAAT	Expression of OptM
PA2070-Hind-R	ACACAAGCTTACCGGATCTGGTAGCTGGAC	Expression of OptM
PA1613-Bam-F	ACACGGATCCGCAATCACGCCATTATACGG	Expression of PA1613
PA1613-Hind-R	ACACAAGCTTCGAGACAGCAGACGACGAG	Expression of PA1613
PA0192-Bam-F	ACACGGATCCTTCGAACCTTTCGCCAGAT	Expression of OptP
PA0192-Hind-R	ACACAAGCTTGTGCTGCATTGCTCATGT	Expression of OptP
PA4837-Bam-F	ACACGGATCCGAGATAGAGCCGGGCATTGTA	Expression of OptC
PA4837-Hind-R	ACACAAGCTTGGCGGTAGAGGGCTCAGTAGT	Expression of OptC
PA2057-Xba-F	ACACTCTAGATGAGTGTCGACCAGGCCCT	Expression of SppR
PA2057-Hind-R	ACACAAGCTTGCTCGTCGTAGAGCGTGATG	Expression of SppR
PA3268-Bam-F	ACACGGATCCGTAATTTTGCGCCCCACTTTT	Expression of OptR
PA3268-Hind-R	ACACAAGCTTCGTCATCGATTGGACTGCAT	Expression of OptR

Table S3 Crystallographic data and refinement statistics

	5NEC
**Data collection**	
Space group	C2
Cell dimensions	
a, *b*, *c* (Å)	164.52, 136.45, 118.95
α, β, γ (°)	90.00, 132.04, 90.00
Resolution (Å)	100-2.3 (2.38-2.3)*
R_{sym} or R_{merge}	0.081 (1.044)
$I / \sigma I$	9.86 (1.23)
Completeness (%)	97.6 (85.1)
Redundancy	3.58 (2.94)
CC half	**-0.569**
**Refinement**	
Resolution (Å)	91.03-2.3
No. reflections	80547
R_{work} / R_{free}	0.223/0.249
No. atoms	
Protein	10073
Ligand/ion	16
Water	65
B-factors	
Protein	57.85 (A)-56.31(B)
Ligand/Water	49.68 (A)-44.23 (B)

TABLE S4 Effect of *optJ* deletion in *piuA* and *pirA* deletion strains of *P. aeruginosa* PAO1

	MIC (mg/liter)				
Strain	BAL	MC-1	ATM	CFD	CAZ
PAO1	1	0.25	4	0.25	2
Δ*piuA*	8	4	4-8	8	2
Δ*pirA*	1	0.5	4	0.5	2
Δ*optJ*	1	0.25	4	0.25	2
Δ*piuA*Δ*optJ*	16	8	4	8	2
Δ*pirA*Δ*optJ*	2	0.5	4	0.5	2
Δ*piuA*Δ*pirA*	16	>16	4	16	2
Δ*piuA*Δ*pirA*Δ*optJ*	32	>16	4	16	2

BAL, BAL30072; ATM, aztreonam; CFD, cefiderocol; CFZ, ceftazidime

Table S5. MICs of siderophore-drug conjugates for PAO1 derivatives determined in iron-sufficient and iron-limited media

Strain	MIC (mg/liter)														
	BAL			MC-1			ATM			CFD			CAZ		
	MHB	MHB-C	M9CAA	MHB	MHB-C	M9CAA	MHB	MHB-C	M9CAA	MHB	MHB-C	M9CAA	MHB	MHB-C	M9CAA
PAO1	1	0.5	0.06	0.5	0.25	0.01	4	4	2	0.5	0.06	0.03	2	2	1
Δ*piuA*	8	1	0.5	8	1	0.25	4	4	4	8	1	0.25	2	2	1
Δ*pirA*	1	0.5	0.06	0.5	0.5	0.01	4	4	2	0.5	0.06	0.03	2	2	1
Δ*piuA*Δ*pirA*	16	4	1	16	2	1	4	4	4	16	0.5	0.25	2	2	1

Iron-sufficient: MHB, Mueller-Hinton broth; iron-limited: MHB-C, chelex-treated MHB; M9CAA, M9 salts based casamino acid medium

References

1. **Van Delden C, Page MG, Köhler T**. 2013. Involvement of Fe uptake systems and AmpC beta-lactamase in susceptibility to the siderophore monosulfactam BAL30072 in *Pseudomonas aeruginosa*. Antimicrob Agents Chemother **57**:2095-2102.
2. **Moynié L, Luscher A, Rolo D, Pletzer D, Tortajada A, Weingart H, Braun Y, Page MG, Naismith JH, Köhler T**. 2017. Structure and Function of the PiuA and PirA Siderophore-Drug Receptors from *Pseudomonas aeruginosa* and *Acinetobacter baumannii*. Antimicrob Agents Chemother **61**.
3. **Thoma S, Schobert M**. 2009. An improved *Escherichia coli* donor strain for diparental mating. FEMS Microbiol Lett **294**:127-132.
4. **Pei J, Kim BH, Grishin NV**. 2008. PROMALS3D: a tool for multiple protein sequence and structure alignments. Nucleic Acids Res **36**:2295-300.
5. **Robert X, Gouet P**. 2014. Deciphering key features in protein structures with the new ENDscript server. Nucleic Acids Res **42**:W320-4.
